# An assessment of the vulnerability of carotid plaques: a comparative study between intraplaque neovascularization and plaque echogenicity

**DOI:** 10.1186/1471-2342-13-13

**Published:** 2013-03-28

**Authors:** Yangyang Zhou, Yan Li, Yang Bai, Ying Chen, Xiaofeng Sun, Yingqiao Zhu, Jiang Wu

**Affiliations:** 1 Department of Neurology, The First Norman Bethune Hospital of Jilin University, Xinmin Street 71#, Chang Chun, 130021, China; 2 Center for Abdominal Ultrasound, The First Norman Bethune Hospital of Jilin University, Chang Chun, China; 3 Center for Neurovascular Ultrasound, The First Norman Bethune Hospital of Jilin University, Chang Chun, China

**Keywords:** Contrast-enhanced ultrasound (CEUS), Plaque vulnerability, Monitoring of microembolic signals (MES), Color Doppler ultrasonography (CDUS)

## Abstract

**Background:**

Carotid plaque echolucency as detected by Color Doppler ultrasonography (CDUS) has been used as a potential marker of plaque vulnerability. However, contrast-enhanced ultrasound (CEUS) has recently been shown to be a valuable method to evaluate the vulnerability and neovascularization within carotid atherosclerotic plaques. The aim of this study was to compare CEUS and CDUS in the assessment of plaque vulnerability using transcranial color Doppler (TCD) monitoring of microembolic signals (MES) as a reference technique.

**Methods:**

A total of 46 subjects with arterial stenosis (≥ 50%) underwent a carotid duplex ultrasound, TCD monitoring of MES and CEUS (SonoVue doses of 2.0 mL) within a span of 3 days. The agreement between the CEUS, CDUS, and MES findings was assessed with a chi-square test. A *p*-value less than 0.05 was considered statistically significant.

**Results:**

Neovascularization was observed in 30 lesions (44.4%). The vascular risk factors for stroke were similar and there were no age or gender differences between the 2 groups. Using CEUS, MES were identified in 2 patients (12.5%) within class 1 (non-neovascularization) as opposed to 15 patients (50.0%) within class 2 (neovascularization) (*p* = 0.023). CDUS revealed no significant differences in the appearance of the MES between the 2 groups (hyperechoic and hypoechoic) (*p* = 0.237).

**Conclusion:**

This study provides preliminary evidence to suggest that intraplaque neovascularization detected by CEUS is associated with the presence of MESs, where as plaque echogenicity on traditional CDUS does not. These findings argue that CEUS may better identify high-risk plaques.

## Background

Internal carotid artery (ICA) disease is frequently observed in ischemic stroke patients. Histological and imaging studies [1-3] have demonstrated that stroke is dependent on the degree of stenosis and the morphological features of the plaque, such as ulcers or fissures. These morphological features can cause a rupture [4] of the plaque and result in embolization, which is known as “vulnerability”. All of these factors should be considered when developing an accurate diagnostic and preventive approach aimed at risk stratification and treatment planning to reduce the incidence and severity of acute cerebrovascular disease. Color Doppler ultrasonography (CDUS) has been the the screening test of choice for assessing carotid atherosclerosis. Echolucency of the Carotid plaque is a valuable marker of the plaque vulnerability [5-7].

However, recently several studies have confirmed the feasibility of using contrast-enhanced ultrasound (CEUS) for the evaluation of neovascularization within carotid atherosclerotic plaques. Furthermore, this technique may also be used to assess the vulnerability of carotid plaques [8-11]. Levovist is an ultrasound contrast agent (BR1; Bracco SpA, Milan, Italy; Definity, Lantheus Medical Imaging) that enables the optimization of technically difficult explorations using a Doppler signal of sufficient intensity and improves the detection of minimal flow rates and slow velocities in severe cases of stenosis.

Previous studies [12,13] demonstrate that echolucent plaques tend to have greater contrast enhancement compared to echogenic plaques. Echolucent plaques are known to exhibit a larger number of vulnerable pathological features and correlate with a higher risk of cerebrovascular events [14]. However, studies to determine the most accurate technique for assessing plaque vulnerability have been limited. The aim of our study was to compare CEUS-detected neovascularization with plaque morphology on CDUS with regards to their correlation with MESs.

## Materials and methods

### Patient group and informed consent

This study was approved by the local ethics committee and written informed consent was obtained from all patients. The following data was recorded from the patient: age, sex, previous symptoms (Transient ischemic attacks (TIA), dysphasia, single limb paresis and amaurosis fugax) and co-morbid risk factors (hypertension, hyperlipidemia, diabetes and smoking habits) (Table 1 and Table 2). Both asymptomatic and symptomatic patients were included.

**Table 1:** Differences in the clinical characteristics of class 1 (non-neovascularization) and class 2 (neovascularization) determined by CEUS

	**Class 1 (n = 16)**	**Class 2 (n = 30)**	**P-value**
Age (years), mean ± SD	64.38 ± 11.32	61.50 ± 6.26	0.186
Men, n (%)	16 (100%)	27 (90%)	0.542
Neurological symptoms*, n (%)	6 (37.5%)	18 (60%)	0.217
Hypertension, n (%)	9 (56.3%)	13 (43.3%)	0.538
Diabetes mellitus, n (%)	3 (18.8%)	9 (30.0%)	0.498
Hypercholesterolemia, n (%)	9 (56.3%)	18 (60.0%)	1.000
Current smoker, n (%)	8 (50.5%)	19 (63.3%)	0.531

*Neurological symptoms include previous transient ischemic attacks (TIA), dysphasia, single limb paresis, and amaurosis fugax.

**Table 2:** Differences in the clinical characteristics of group 1 (hyperechoic) and group 2 (hypoechoic) determined by CDUS

	**Group 1 (n = 28)**	**Group 2 (n = 18)**	**P-value**
Age (years), mean ± SD	62.22 ± 6.31	62.96 ± 9.54	0.456
Men, n (%)	26 (92.9%)	17 (94.4%)	1.000
Neurological symptoms*, n (%)	16 (57.1%)	8 (44.4%)	0.295
Hypertension, n (%)	17 (60.7%)	10 (55.6%)	0.767
Diabetes mellitus, n (%)	8 (28.5%)	5 (27.8%)	1.000
Hypercholesterolemia, n (%)	15 (53.6%)	12 (66.7%)	0.541
Current smoker, n (%)	18 (64.3%)	13 (72.2%)	0.749

*Neurological symptoms include previous transient ischemic attacks (TIA), dysphasia, single limb paresis, and amaurosis fugax.

Between March 2011 and March 2012, 54 subjects with arterial stenosis (≥50%) underwent CDUS (PSV_ICA_≥125 cm/s and visible plaque [15]) at the First Norman Bethune Hospital of Jilin University. In addition, TCD monitoring of MES and CEUS examinations were performed within 3 days by two independent researchers (Y.L. and Y.B.). MES monitoring was performed by two neuroradiologists (Y-Q.X and Y.C.). Patients with any of the following conditions were excluded: [1] complete ICA occlusion or <50% stenosis based on CDUS; [2] evidence of cardioembolism, such as atrial fibrillation, mechanical valve replacement, left atrial or left ventricular thrombus, bacterial endocarditis, or recent myocardial infarction; [3] ipsilateral stenosis of the middle cerebral artery (MCA) or intracranial internal carotid artery in the TCD; [4] a poor temporal window; or [5] poor image quality of the vessel wall or lumen. Therefore, data from remaining 46 patients (43 male and 3 women) with satisfactory image quality were analyzed.

### Color Doppler ultrasonography (CDUS)

We used an ultrasound Philips iU22 system (Philips Healthcare Solutions, Bothell, WA, USA) equipped with an L-9-3 linear-array transducer. The instrument was operated by 2 experienced readers (Y-YZ and YC), who were blinded to all of the clinical laboratory findings and other imaging data.

The maximal thickness of the lesion located at the bifurcation and proximal to the bifurcation was assessed as a continuous variable and measured from the anterior, lateral and cross-sectional scanning plane using a longitudinal image from the media-adventitia to the intima-lumen boundaries. The B-mode settings were adjusted to optimize the quality of the gray-scale images and the pulse repetition frequency (PRF) used with the color Doppler flow imaging was adjusted according to the flow velocity.

The characteristics of the plaques were described according to the modified Gray Weale classification [16]. The lesion echogenicity was classified into group 1 (uniformly hyperechoic or predominantly (>50%) hyperechoic) or group 2 (uniformly hypoechoic or predominantly (>50%) hypoechoic). All results were agreed upon by at least two experienced neuroradiologists.

### Contrast-enhanced ultrasound (CEUS)

A contrast-enhanced ultrasound examination was performed using an Acuson Sequoia 512 imaging system (Siemens, Mountain View, CA, USA) with a 2-MHz transducer by 2 experienced readers (Y.B. and Y.L.), who were blinded to all of the clinical laboratory findings and other imaging data. Disagreements between the readers were settled by a consensus reading. The patients were placed in a supine position. A 5-mL solution was prepared from 1 mL of the activated contrast agent (BR1; Bracco SpA, Milan, Italy; Definity, Lantheus Medical Imaging) diluted in 4 mL of saline. An initial bolus injection was quickly performed. The second injection was performed slowly and was followed by 5 mL of normal saline to flush out the contrast from the vein. The time gap between the injections was approximately 3 minutes. The contrast-enhanced ultrasound imaging application included a low mechanical index (0.07) to avoid early bubble destruction and harmonics with pulse inversion to optimize the depiction of the IV contrast agent and minimize echoes from the surrounding tissues. Cine loops were recorded for 5 heart cycles, starting from the time in which the contrast agent could be observed in the carotid lumen. Following the infusion of the ultrasound contrast agent, the lumen of the carotid artery was enhanced, resulting in visualization of enhanced plaque luminal morphology. The presence of blood flow “activity” was identified on the basis of the dynamic movement of the echogenic reflectors (microspheres) in the intraplaque microvessels.

Intraplaque neovascularization (contrast agent enhancement) was categorized using a modified grading scale and classified as class 1 (non-neovascularization) or class 2 (neovascularization).

### TCD ultrasound examination

MES monitoring was performed by two experienced neuroradiologists (Y-YZ and YC) with a TCD machine (EME TC8080; Nicolet, Madison, WI, USA) with a 2-MHz transducer. The patients were placed in the supine position and bilateral MCA recordings were obtained for 30 minutes at a depth of 44–60 mm. The MES were identified on the basis of Doppler waves obtained from the MCA ipsilateral to the side of the ICA stenosis. The following definitions for emboli signals were used: typical, visible, and audible (click, chirp, whistle). Short-duration, high-intensity signals within the Doppler flow spectrum occurred at random intervals during the cardiac cycle. Signals were defined at 6 dB above the background threshold on the basis of standard consensus criteria described in previous studies. The presence of MES was assessed by an independent expert reader (Y-QX), who was blinded to all of the clinical laboratory findings and other imaging data.

### Database and statistical analysis

All of the data were analyzed with SPSS 17.0. The results are expressed as the mean value and standard deviation (SD) for each measurement. Categorical variables were assessed using the chi-square test. A *p*-value of less than 0.05 was considered statistically significant.

## Results

Forty-six patients (43 men and 3 women) with satisfactory image quality were analyzed. The differences in the clinical characteristics are reported in Table 1 and Table 2.

**Figure 1:**
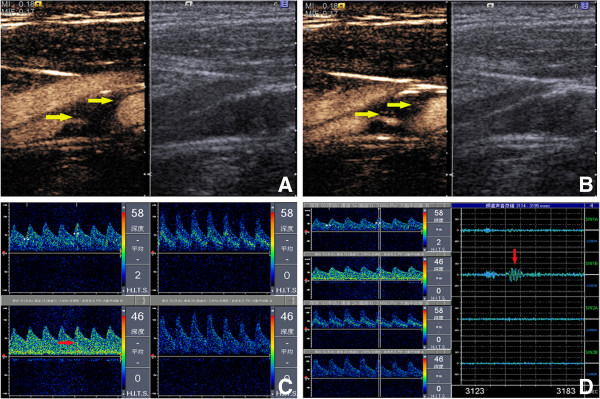
**A 56 year-old female patient with TIA.** CEUS (**A** and **B)** detected 2 consecutive frames of intra-plaque neovascularization (yellow arrow). MES (red arrow) was detected in the ipsilateral middle cerebral artery (**C** and **D**).

CEUS revealed neovascularization in 30 patients (44.4%). The stroke vascular risk factors were similar between groups, and there were no age or gender differences between the 2 classes (Table 1). MES were observed in 2 patients (12.5%) within class 1 (non-neovascularization) and in 15 patients (50.0%) within class 2 (neovascularization) (*p* = 0.0230) (Table 3, Figure 1).

**Table 3:** Comparison of the MES measurements (mean D) of class 1 (non-neovascularization) and class 2 (neovascularization) determined by CEUS

	**Class 1 (n = 16)**	**Class 2 (n = 30)**	**P-value**
MES (z), n (%)	2 (12.5)	15 (50.0)	0.023

Using CDUS, 28 patients were identified in group 1 (hyperechoic), and 18 were identified in group 2 (hypoechoic). Stroke vascular risk factors, age and gender were similar between the 2 groups (Table 2). Moreover, no significant differences were observed in the appearance of the MES between the 2 groups (*p* = 0.2368) (Table 4).

**Table 4:** Comparison of the MES measurements (mean D) of group 1 (hyperechoic) and group 2 (hypoechoic) determined by CDUS

	**Group 1(n = 28)**	**Group 2 (n = 18)**	**P-value**
MES (z), n (%)	12 (42.9)	5 (27.8)	0.237

## Discussion

In this study, we examined the relationship between CEUS and CDUS characteristics of the carotid plaque with special reference to MES. We found an association between MES monitoring and the degree of contrast-agent enhancement using ultrasound imaging (*p*=0.0230). However, we did not observe a significant association between the MES results and CDUS properties (*p* = 0.2368). Although plaque echolucency is a marker of high-risk lesions (rupture prone plaques), our findings indicate that CEUS appears to be more accurate at assessing plaque vulnerability.

CDUS has replaced digital subtraction angiograph for the diagnosis of carotid stenosis, in part because CDUS provides enhanced definition of plaque morphology [17,18]. Several studies [5,14,19-21] have demonstrated that echogenic plaques are well-established markers of high-risk lesions and are associated with the presence of neurological symptoms and the development of future strokes in previously symptomatic individuals. Furthermore, echogenic plaques also coincide with the occurrence of acute coronary syndromes. Several studies have reported [22-26] that echogenic plaques are prone to rupture due to their increased lipid content and macrophage density as well as intraplaque hemorrhage; in addition, increased plasma and low-density lipoprotein cholesterol levels make them vulnerable and prone to ulceration and embolization.

Neovascularization is considered an important feature in plaque development and vulnerability and is triggered by inflammation and hemorrhage [8,27,28]. The vulnerability of the neovasculature to rupture increases the risk of cerebral emboli. Several pathological studies [28-30] have confirmed that plaque rupture is strongly associated with the presence and degree of neovascularization within the plaque.

Intraplaque microvessels (angiogenesis) within the atherosclerotic lesions arise mainly from the adventitial vasa vasorum. Extension of the vasa vasorum to the full thickness of the media and intima of atherosclerotic segments represents pathological neovascularization, which is stimulated by plaque hypoxia, reactive oxygen species, hypoxia-inducible factor signaling and inflammation [31,32].

Feinstein et al. [33] and Assaf Hoogi et al. [11] compared the results of CEUS with histological characteristics. Their findings revealed that contrast enhancement within the plaque is correlated with a higher number of microvessels. The studies of Staub *et al*. [10] and Faggioli *et al*. [34] have indicated the feasibility of using CEUS to depict neovascularization within the carotid plaque to facilitate the further stratification of the risk of rupture of carotid artery lesions. Thus, CEUS has been proposed as a method to preoperatively identify vulnerable plaques.

Consistent with the data obtained in previous reports [12,13], neovascularization visualized using CEUS is correlated with the morphological features of plaque vulnerability, including echogenic plaques, as a marker of high risk lesions.

Coli *et al.*[12] reported that carotid plaque contrast agent enhancement correlated with echogenic plaques (*p* = 0.001) and is associated with the histological density of neovessels. Interestingly, intraplaque neovascularization in CEUS images correlated well with histological microvessel density rather than plaque echolucency suggesting that low echo intensity is not correlated with the histological density of the vasa vasorum. Thus CEUS is a more specific imaging modality to identify highly vascularized and inflamed vulnerable lesions as compared to standard CDUS in isolation.

Our observations strongly indicate a positive relationship between neovascularization in plaques and MES while there is a poor correlation between plaque echolucency and MES. Embolism is an important mechanism of cerebral infarcts in patients with ICA stenosis [35]. The detection of cerebral microembolisms by transcranial Doppler sonography may permit the definition of a high-risk subgroup among patients with asymptomatic high-grade internal carotid artery stenosis [36]. To the best of our knowledge, this is the first study to explore neovascularization in stroke patients with ICA stenosis using MES and CEUS.

There were some limitations in this study. The first limitation was that the pilot study was conducted with a small sample size. Second, we used a semi-quantitative approach to evaluate the contrast-agent enhancement; however, this limitation does not alter our observations or conclusions. This quantitative method needs to be further investigated. Finally, several patients could not be examined because of an inadequate insonation window during the TCD monitoring that prevented further analysis. Future studies in larger populations are required to validate the results of the present study. Moreover, prospective clinical studies are also needed to evaluate the use of contrast-enhanced ultrasound imaging of plaque neovascularization to assess the risk of cerebrovascular events and to monitor the effects of anti-atherosclerotic therapies.

## Conclusions

Intraplaque neovascularization detected by CEUS but not plaque echolucency is correlated with MES, suggesting that CEUS may provide valuable information about plaque risk stratification and may be an accurate method for assessing vulnerable plaques beyond the echogenicity of CDUS.

## Competing interests

The authors declare that there are no competing interests.

## Authors’ contributions

YZ and YX participated in the design of the study, performed the statistical analysis, participated in the sequence alignment, CDUS, and MES studies, and drafted the manuscript. YL, YB, XS, and YZ performed the CEUS studies. YC participated in the CDUS and MES studies. YX and JW developed the concept of the study and participated in its design and coordination. All authors have read and approved the final manuscript.

## Pre-publication history

The pre-publication history for this paper can be accessed here:

http://www.biomedcentral.com/1471-2342/13/13/prepub
